# Assessment of Social Support and Quitting Smoking in an Online Community Forum: Study Involving Content Analysis

**DOI:** 10.2196/34429

**Published:** 2022-01-13

**Authors:** Laura Struik, Shaheer Khan, Artem Assoiants, Ramona H Sharma

**Affiliations:** 1 School of Nursing University of British Columbia Kelowna, BC Canada; 2 School of Health and Exercise Science University of British Columbia Kelowna, BC Canada; 3 PH1 Research Vancouver, BC Canada; 4 School of Social Work University of British Columbia Kelowna, BC Canada

**Keywords:** qualitative research, smoking cessation, social media, social support, smoking, tobacco use, tobacco, online forum

## Abstract

**Background:**

A key factor in successfully reducing and quitting smoking, as well as preventing smoking relapse is access to and engagement with social support. Recent technological advances have made it possible for smokers to access social support via online community forums. While community forums associated with smoking cessation interventions are now common practice, there is a gap in understanding how and when the different types of social support identified by Cutrona and Suhr (1992) (emotional, esteem, informational, tangible, and network) are exchanged on such forums. Community forums that entail “superusers” (a key marker of a successful forum), like QuitNow, are ripe for exploring and leveraging promising social support exchanges on these platforms.

**Objective:**

The purpose of this study was to characterize the posts made on the QuitNow community forum at different stages in the quit journey, and determine when and how the social support constructs are present within the posts.

**Methods:**

A total of 506 posts (including original and response posts) were collected. Using conventional content analysis, the original posts were coded inductively to generate categories and subcategories, and the responses were coded deductively according to the 5 types of social support. Data were analyzed using Microsoft Excel software.

**Results:**

Overall, individuals were most heavily engaged on the forum during the first month of quitting, which then tapered off in the subsequent months. In relation to the original posts, the majority of them fit into the categories of sharing quit successes, quit struggles, updates, quit strategies, and desires to quit. Asking for advice and describing smoke-free benefits were the least represented categories. In relation to the responses, encouragement (emotional), compliment (esteem), and suggestion/advice (informational) consistently remained the most prominent types of support throughout all quit stages. Companionship (network) maintained a steady downward trajectory over time.

**Conclusions:**

The findings of this study highlight the complexity of how and when different types of social support are exchanged on the QuitNow community forum. These findings provide directions for how social support can be more strategically employed and leveraged in these online contexts to support smoking cessation.

## Introduction

### Background

Tobacco use continues to be the number one cause of preventable disease and death around the world, including North America [1,2]. Approximately 12% of Canadians (3.7 million) and 14% of Americans (40 million) smoke cigarettes [3,4]. More than 50% of these smokers in both countries report a desire to quit [5,6], and 54% of Americans and 42% of Canadians who smoke stated that they tried to quit in the last year [3]. Although most of the smoking population has stated a desire to quit or has attempted to quit, a little over 6% of Americans who smoke were able to successfully quit in 2018 [5]. There is a similar proportion in Canada, where only 6% of former smokers quit within the last year [6].

There are many cessation services and interventions now available to support and enhance the cessation efforts of individuals. A recommended strategy in North American best practice guidelines for smoking cessation interventions and services is the inclusion and recommendation of social support [7,8]. Social support is defined as interpersonal communication that is characterized by the exchange of informational and emotional resources among and across networks [9]. In addition to receiving support from clinicians and tobacco specialists, engagement with social support communities/networks has been consistently found to be a key factor in successfully reducing and quitting smoking, as well as preventing relapse [10-14]. Because of the benefits that social support offers, smokers participating in behavioral interventions for smoking cessation are commonly advised to seek social support [15-19]. Recent technological advances have made it possible for social support to be available online through community forums associated with cessation services and interventions, enabling significant reach to a variety of populations [20]. While community forums associated with smoking cessation interventions are now common, there is a gap in understanding the nature of social support exchanges on such forums.

Social support has been broken down into the following 5 different types according to Cutrona and Suhr [9]: emotional, esteem, informational, tangible, and network support (Multimedia Appendix 1). This social support framework is useful for understanding what types of social support are most useful in different behavioral contexts [21]. However, little is known about what types of social support are needed and when, especially when it comes to a behavior like smoking, which is often not a linear process. Understanding what types of social support are provided at different time points throughout the quitting trajectory would provide both service providers and end-users with specific directions for maximizing social support associated with an intervention. Community forums for smoking cessation are ripe platforms for investigating the different types of social support exchanges at different points in time.

A key marker of successful community forums on health-related topics is the engagement of superusers [22,23]. Superusers are voluntary users who remain disproportionately engaged and essentially serve to keep the forum “alive” through their active engagement over a long-term timeframe [22]. Superusers play a critical role in generating and exchanging content, support, and advice, and prompting discussions [22]. QuitNow is an example of a community forum for smoking cessation that includes superusers. However, little is known about the nature of the social support provided on these forums in general, and especially on forums that entail superusers. Unpacking how social support is activated on a successful forum will assist in leveraging its success, as well as enhancing other aspects of cessation services and interventions. The purpose of this study, therefore, was to characterize the posts made on the QuitNow community forum at different stages in the quit journey, and determine when and how the social support constructs are present within the posts.

### QuitNow

QuitNow is British Columbia’s free online smoking cessation service delivered by the British Columbia Lung Association on behalf of the Government of British Columbia. It is a customized program for British Columbia residents who are looking to quit or reduce tobacco use, including smoking. On the website, there are resources for different stages of quitting (eg, thinking about quitting, preparing to quit, and staying quit). There are also resources for health professionals, families, and friends to support smokers who want to quit. Individuals are encouraged to create an account with QuitNow, which gives them access to free quit coaching, live chat with a quit coach, and a community forum. A quit coach is an individual trained and paid to provide cessation support to individuals trying to quit smoking. The community forum is the primary social support feature of QuitNow and is moderated by quit coaches. The content is publicly available, but engagement with the forum requires registration. Given that the posts examined for this study are publicly available, in consultation with the Behavioral Research Ethics Board and the University of British Columbia’s Okanagan campus, it was agreed that ethical approval was not required.

## Methods

### Data Collection

For this study, a total of 506 QuitNow community forum posts spanning the month of December 2019 were collected and entered into Excel for conventional content analysis. These posts were collected in reverse chronological order so that the most recent activity on the QuitNow community forum page was represented. Sampling was driven by saturation of codes, where posts were collected until no new categories or subcategories were identified [24].

### Data Analysis

Using conventional content analysis [25], we developed both inductive and deductive categories to identify what types of social support smokers need at different stages in their quit journey. First, we inductively assigned codes to categorize the original posts, noting the user’s stage in quitting. The frequency of posts per category was investigated. Next, 5 quit stages were identified and classified as follows: (1) before quitting, (2) 1st month, (3) 2nd month, (4) 3+ months, and (5) relapse. The variability of user engagement over time and per quit stage was examined. Next, we applied the social support framework developed by Cutrona and Suhr [9] to deductively code the responses to see what types of social support were given in response to the original posts. We also inductively coded responses that did not fit within the definitions of the social support constructs. Two authors (LS and AA) engaged in 3 collaborative coding sessions via UBC Zoom to code 75 posts. Through these collaborative coding sessions, the authors developed a coding legend and assigned codes directly onto the Excel document. As the authors applied the coding legend to the posts, any discrepancies in coding were brought up in real time. The coding legend was revised, and the parameters for what is included in a code were revisited for enhanced clarity. The 2nd coder (AA) then independently coded the remaining posts, consulting with the 1st author regularly as needed. Finally, 2 different authors (SK and RS) developed analytic tables and charts to offer a visual display of what types of supports were provided and when in the quit smoking trajectory as discussed by users on the QuitNow forum.

## Results

### Users

In total, 89 unique users are represented in this sample of posts, with 413 posts made by QuitNow users and 93 made by a Quit coach.

### Posts

Of the 506 posts, 76 were original posts and 430 were responses or subresponses to these posts. The average number of responses per original post was 6, with a range of 4 to 8 responses. Each post contained an average of 46.27 words.

### Original Posts

The data within the 76 original posts were coded (n=145); these codes were subsequently classified into the following 7 categories: (1) quit desires, (2) quit struggles, (3) updates, (4) successes, (5) quit strategies, (6) advice requests, and (7) smoke-free benefits (Table 1).

The frequency of posts per category was examined. The greatest numbers of original posts were in the categories *successes* and *quit struggles*, followed by *updates*, *quit strategies*, and *desires to quit*. *Advice requests* and *smoke-free benefits* had the lowest numbers of posts.

**Figure 1:**
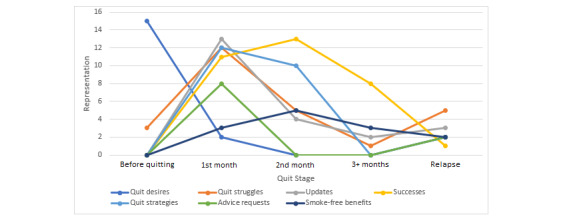
Trajectory of post category representation throughout the quit journey.

Next, variability in user engagement over time was investigated. The 1st month entailed the most engagement, followed by the 2nd month (Table 2). During the before quitting stage, users primarily posted about their quit desires. During the 1st month of quitting, all categories other than *quit desires* and *smoke-free benefits* were almost equally represented. During the 2nd month, *successes* and *quit strategies* were the most commonly posted categories. In 3+ months of their quit journeys, users most frequently shared *successes*. Finally, during the relapse stage, sharing struggles was most common. Figure 1 displays a visual representation of the variability in category representation throughout the quit journey.

**Table 1:** Categories of original posts.

Category	Description	Exemplary quote	Total value (N=145), n
Quit desires	Sharing their desires, plans, or intentions to quit.	*Been battling for a while with quitting and starting quitting and starting. But ready to just call it quits. I hate smoking and no longer want it in my life or in my thoughts. It's time!*	19
Quit struggles	Sharing past, current, or anticipated struggles that make quitting more difficult.	*I have tried numerous times to quit. I was vaping to help cut down, but with all the problems people are having from vaping, I stopped using my vape. But I smoke more now.*	26
Updates	Sharing an update on their quitting status.	*Hello. This is day 8 and still doing well, I am using the patch and its working as well.*	22
Successes	Sharing their experienced successes.	*I’ve made it 3 weeks! Longest smoke-free run since starting when I was 19. I’ve tried seriously quitting since January of this year, & kept hitting a wall at 3-days, even made 2 weeks in April, but got confident and had “one puff” at my birthday party, and you know how that turned out. Although I don’t always post, I do look to this community daily for inspiration. Thanks to all who share their experiences, I can relate to so many of you. :)*	33
Quit strategies	Sharing tips and strategies that worked for them, and strategies they will use.	*As an aside, if you don't exercise regularly, I highly recommend you try doing that now. It does wonders to blow off steam and reduce withdrawal anxiety for me. Maybe it will for you, too.*	24
Advice requests	Asking for advice to navigate circumstances they are experiencing.	*I bought smokes yesterday because I got stressed and anxious. I think I will get the patch. What ways are you all using to quit?*	10
Smoke-free benefits	Sharing benefits of being smoke free.	*My favorite thing about being quit is not being a slave to this addiction anymore. I am mindful [of] all the times I would have been thinking “this is when I’d go for a smoke” or “do I have enough cigarettes till tomorrow?” or “Where can I smoke?” or “when will I have a chance to smoke?”.... and the list goes on. What a giant, toxic waste of time smoking is!*	11

**Table 2:** Representation of categories at each quit stage.

Stage and category		Representation (N=145 posts)
**Before quitting (18 posts), n (%)**		
	Quit desires	15 (83.3)
	Quit struggles	3 (16.6)
**1st month (61 posts), n (%)**		
	Quit desires	2 (3.3)
	Quit struggles	12 (19.6)
	Updates	13 (17.5)
	Successes	11 (18.0)
	Quit strategies	12 (19.6)
	Advice requests	8 (13.1)
	Smoke-free benefits	3 (4.9)
**2nd month (37 posts), n (%)**		
	Quit struggles	5 (13.5)
	Updates	4 (10.8)
	Successes	13 (35.1)
	Quit strategies	10 (27.0)
	Smoke-free benefits	5 (13.5)
**3+ months (14 posts), n (%)**		
	Quit struggles	1 (7.1)
	Updates	2 (14.3)
	Successes	8 (57.1)
	Smoke-free benefits	3 (21.4)
**Relapse (15 posts), n (%)**		
	Quit benefits	2 (13.3)
	Quit struggles	5 (33.3)
	Updates	3 (20.0)
	Successes	1 (6.7)
	Quit strategies	2 (13.3)
	Advice requests	2 (13.3)

### Response Posts

In relation to the 430 responses or subresponses to the original posts, 1100 codes were assigned and broken down into the following 5 social support categories: (1) emotional support, (2) esteem support, (3) informational support, (4) tangible support, and (5) network support, as well as an “other” category, the latter of which was inductively derived (Table 3). With the exception of tangible support, the remaining 4 social support constructs were present within the responses. Emotional, esteem, and informational support were the most prominent social support categories present. Network support was the least present. Most posts contained multiple social support constructs, which are exemplified in the following quote:

….I really admire you for remaining so positive and so proud of you for not giving up (**compliment under esteem support**). I know you'll beat the nicodemon and send him back to where the sun don't shine (**encouragement under emotional support**). Just look at him in the eye and tell him “You're not in control anymore, I am” (**suggestion under information support**). 30 days is awesome and quitting is one of the best gifts you can ever give yourself (**compliment under esteem support**).

Out of the possible 26 social support subcategories, 14 were present within the responses. Encouragement under emotional support and compliments under esteem support were the most strongly represented at 20.9% (n=230) and 20.4% (n=224) of the 1100 codes, respectively. Suggestion/advice under informational support was the next most common at 15.6% (n=172). The least represented subcategories fell under emotional, esteem, and informational support (sympathy, relief from guilt, and teaching, respectively) at 0.9% (n=10) each.

Parallel to how the number of original posts was the highest during the 1st month, the numbers of user responses and subresponses were also at their highest during this time (Table 4). Additionally, responses were also very high during the before quitting stage as well as during the 2nd month. Overall, encouragement, compliment, and suggestion/advice consistently remained the most prominent types of support throughout all the stages. Companionship maintained a steady downward trajectory, with the most companionship posts during the before quitting stage and the least during the 3+ months stage.

**Figure 2:**
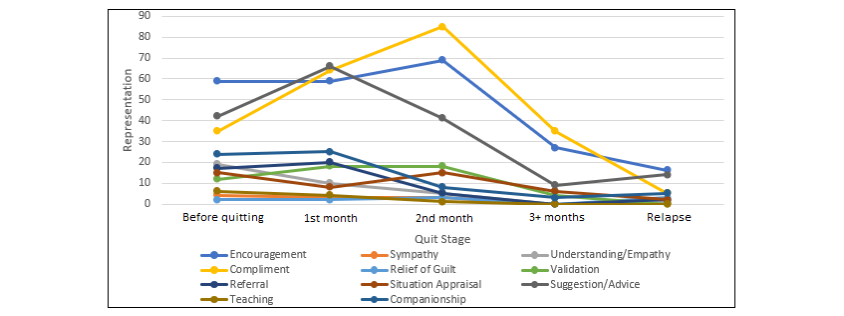
Trend of social support response representation throughout each quit stage.

During the before quitting stage, encouragement, compliment, and suggestion/advice were the most prominent types of support offered to users. During the 1st month, these were also the most prominent but in the reverse order whereby suggestion/advice was the most prominent, followed by compliment and then encouragement. During the 2nd and 3rd months, again, these were the most prominent types of support but with compliment being the most prominent, followed by encouragement and then suggestion/advice. During the relapse stage, encouragement and suggestion/advice were the most prominent, and were equally followed by compliment and companionship. The above trends can be visualized in Figure 2.

**Table 3:** Social support responses to original posts.

Code and subcode		Exemplary quote	Value (N=1100), n (%)
**Emotional (n=275)**			
	Encouragement	*So glad you're trying again. I hope this is the one for you! Good luck, and keep strong, you can do this!*	230 (20.9)
	Sympathy	*I'm sorry to hear that you were allergic to the patch...Did you have a chance to discuss with your doctor or pharmacist?*	10 (0.9)
	Understanding/empathy	*I know what you are going through. I was a two pack a day smoker and it was killing me as well, it has been ten days since my last smoke and I was smoking for 30 years.*	35 (3.2)
**Esteem (n=286)**			
	Compliment	*With your commitment to quit and all these great strategies, you seem well prepared and equipped to fight the cravings and conquer this nicotine addiction!*	224 (20.4)
	Relief of guilt	*You can only do what you can, try again, and one of these times it will stick for good. Don’t be too hard on yourself.*	10 (0.9)
	Validation	*Quitting is difficult and most people try several times before they are successful.*	52 (4.7)
**Informational (n=283)**			
	Referral	*BC Quit Now helpline is always available to help you plan and provided you with tips and tricks to make your quit successful. If you need extra support, give us a call, we would be happy to help you 1-877-455-2233.*	44 (4.0)
	Situation appraisal	*Quitting is a selfish thing that you do for yourself but the benefits carry over to all the other people in your life!*	56 (5.1)
	Suggestion/advice	*I'm using the patch and lozenge and went down a step on the patch last week. Slow and steady, right? Remember - this is a marathon, not a sprint.*	172 (15.6)
	Teaching	*Many studies have shown that physical activities such as aerobic exercise help reduce the urges to smoke and decrease the withdrawal symptoms and cravings for cigarettes.*	11 (1.0)
**Network (n=85)**			
	Companionship	*There's lots of support here on the forum; take time to read the others and their quit journey. You can also call a quit coach to help you make a plan. Good luck!*	85 (7.7)
**Other (n=191)**			
	Appreciation	*Awe thanks for your support....I am hanging in there and I know with time it will get easier!*	82 (7.5)
	Happy holidays	*Hope you have a very Merry Christmas and Happy New Year. Hugs.*	89 (8.1)

**Table 4:** Social support responses represented in each quit stage.

Stage and category			Representation (N=1100 posts)
**Before quitting (235 posts), n (%)**			
	Encouragement	59 (25.1)	
	Sympathy	4 (1.7)	
	Understanding/empathy	19 (8.1)	
	Compliment	35 (14.9)	
	Relief of guilt	2 (0.9)	
	Validation	12 (5.1)	
	Referral	17 (7.2)	
	Situation appraisal	15 (6.4)	
	Suggestion/advice	42 (17.9)	
	Teaching	6 (2.6)	
	Companionship	24 (10.2)	
**1st month (279 posts), n (%)**			
	Encouragement	59 (21.1)	
	Sympathy	3 (1.1)	
	Understanding/empathy	10 (3.6)	
	Compliment	64 (22.9)	
	Relief of guilt	2 (0.7)	
	Validation	18 (6.5)	
	Referral	20 (7.2)	
	Situation appraisal	8 (2.9)	
	Suggestion/advice	66 (23.7)	
	Teaching	4 (1.4)	
	Companionship	25 (9.0)	
**2nd month (253 posts), n (%)**			
	Encouragement	69 (27.3)	
	Sympathy	3 (1.2)	
	Understanding/empathy	5 (2.0)	
	Compliment	85 (33.6)	
	Relief of guilt	3 (1.2)	
	Validation	18 (7.1)	
	Referral	5 (2.0)	
	Situation appraisal	15 (5.9)	
	Suggestion/advice	41 (16.2)	
	Teaching	1 (0.4)	
	Companionship	8 (3.2)	
**3+ months (84 posts), n (%)**			
	Encouragement	27 (32.1)	
	Compliment	35 (41.7)	
	Validation	4 (4.8)	
	Situation appraisal	6 (7.1)	
	Suggestion/advice	9 (10.7)	
	Companionship	3 (3.6)	
**Relapse (48 posts), n (%)**			
	Encouragement	16 (33.3)	
	Understanding/empathy	1 (2.1)	
	Compliment	5 (10.4)	
	Relief of guilt	3 (6.3)	
	Referral	2 (4.2)	
	Situation appraisal	2 (4.2)	
	Suggestion/advice	14 (29.2)	
	Companionship	5 (10.4)	

## Discussion

### Principal Findings

This study highlights the complexity of how and when different types of social support are exchanged among individuals quitting smoking on the QuitNow online community forum. Application of Catrona and Suhr’s [9] social support framework to such a successful online community forum enabled the ability to identify specific ways in which individuals support each other at different stages in the quit process. This extends what was previously known about the role of social support on online forums for quitting smoking. Previous work has examined the overall purpose of social support exchange online [26], the different types of social support provided [27], and the impact of different types of social support on communication patterns for smoking cessation [28]. In this study, by unpacking how specific types of social support are most fruitful during different stages of quitting, the findings provide directions for how social support can be more strategically employed in these online contexts to support smoking cessation.

### User Engagement

The highest and most varied engagement on the forum occurred throughout the 1st month of quitting. This coincides with the biological processes that occur during cessation. Many users face withdrawal symptoms in the short term following an attempt to quit [6]. These symptoms of withdrawal will last 2 to 4 weeks, with symptoms typically emerging within the initial couple of days following quitting, and peak in the 1st week [6]. To manage withdrawal, individuals often rely on the support of others, such as through community forums for smoking cessation. Our finding that people asked for advice mostly in the 1st month or during relapse may be an example of users seeking more strategies and support when their withdrawal symptoms are the highest. Given that engagement on the forum parallels these biological processes, this carries implications for how social support may be optimized to assist individuals in getting through withdrawal symptoms (eg, strategically offering advice, distraction tips, what to expect, etc) during that 1st month.

We also found that user engagement steadily decreases after 2 to 3 months. Again, this likely reflects the nicotine dependence trajectory, wherein receptors in the brain fully adapt to nonsmoking after 3 months due to organic reversals in brain matter; nicotine-related deficits in brain dopamine are often a consequence of chronic smoking that revert to baseline 3 months after quitting [29]. Additionally, individuals may feel confident in their quit status by this time, so the need for support consequentially drops.

### Original Posts

Forum users increasingly shared their cessation journey successes and strategies with others during the 1st and 2nd months of quitting. This may suggest that, by this time, individuals feel confident enough to share their successes and feel that they have enough personal credibility to offer advice. Previous literature has found that individuals are often reluctant to share their successes too early due to a fear of failure and a lack of confidence in their ability, which is consistent with these findings [30]. In keeping with these findings, individuals on the forum appear to be less likely to share their success unless they are confident in their ability to sustain their behavior change. These findings are significant in that sharing successes may serve as an effective measure of quit success and self-efficacy when evaluating the impact of smoking cessation forums.

In response to individuals sharing successes, others primarily provide esteem support, possibly further boosting their self-efficacy, a key ingredient to behavior change overall [31] and to maintaining a successful quit status and preventing relapse [32]. While the findings of this study are helpful from a cessation success perspective, there may be smaller successes worth sharing (eg, overcoming the 1st couple of days), wherein individuals can receive that esteem support earlier, potentially retaining individuals in their quit trajectory due to a boost in their self-efficacy. While more research is needed, calls to invite individuals to share their “smaller” successes may prove beneficial.

### Social Support Responses

Overall, the types of social support that were most consistently present included emotional and esteem support. This is in keeping with previous research, whereby nurturant support (including emotional and esteem support) was found to be most common in responses to original posts [33]. Tangible support was not present in this analysis, possibly due to the limitations of the forum. One way to provide tangible support is through active participation in an activity, like quitting with a quit buddy. This was not enabled via the forum, but may be a worthwhile area for exploration.

It is important to note that companionship demonstrated to have a steady downward trajectory over time; it was most prevalent before quitting but less so over time, with the exception of relapse. This is significant as it exemplifies the importance of letting people know that they are in this together at the beginning and particularly before the quitting process, and once again speaks to the importance of a tailored individualistic intervention process. Simply, individuals need to feel like they are not alone as they embark on the journey to quit using nicotine.

### Recommendations and Future Research

The results of this study encompass several recommendations that could benefit QuitNow, as well as smoking cessation community forums at large. One recommendation is to provide community forum users, as well as family and friends, with a tip sheet based on the findings (eg, emphasizing understanding and companionship at the before quitting stage; emphasizing encouragement and compliments during active quitting; and helping them reappraise the situation/offer relief from guilt during relapse). In addition, community forums may benefit from having discussion tags, whereby users can decide what types of content they are most interested in viewing. For example, a user who is thinking about quitting might want to know what the 1st month is like and may filter forum content through a “1st month” tag. Finally, the results have implications for the implementation of online programs in which community forums are embedded. For example, now that we know companionship is key at the beginning of the quit journey, the affiliated website would benefit from emphasizing the presence of social supports (including the forum), which may subsequently retain individuals in the program. As soon as a new user joins a forum, a quit coach could send a text or email message about being there for them and referring them to the community forum (offering companionship early on).

Future research is needed to assess how changes made to the QuitNow forum as a result of this study’s findings impact engagement and cessation outcomes among users. For example, do more strategic prompts to use the forum and to post about smaller successes result in more month-to-month engagement (eg, more original posts and responses)? Moreover, it would be interesting to determine how different groups are engaging on the forum (eg, are certain populations staying in a particular quit stage longer?). This would carry implications for more tailored support for different groups. It would also be worthwhile assessing whether posting about big successes (eg, 2 months smoke-free) can be an effective measure and possible predictor of abstinence. This could potentially lend to an innovative and nonintrusive way of determining the success of a forum in supporting cessation.

### Limitations and Strengths

This study has several limitations. First, the frequency, duration, and number of cigarettes smoked among the various forum users could not be collected. These factors may play a role in shaping individual behaviors during a cessation attempt and/or journey. Second, we did not analyze intersections of identity (eg, gender, race/ethnicity, orientation, education level, or socioeconomic status) and their impact on how support was exchanged on the forum. In this regard, we were not able to collect data on nuances regarding what type of support is most beneficial to whom at different points in the quit trajectory. Third, it may be possible that some forum users did not update their quit status on the forum. Finally, the data collected for this study represent a specific point in time (leading up to Christmas and the new year), which may influence the type of communication and motivation of the users, and possibly atypical engagement on the forum. While this may be a limitation, it is also a strength in that these posts reflect the most recent and up-to-date posts at the time of the analysis. Another strength of the study is that data available in real-time were collected to gather insights into user engagement. Finally, this study encompassed the use of inductive qualitative methods to identify key themes and a theoretical framework to understand social support exchanges on the forum. This lends to a strong evidence base from which to move forward with recommendations.

### Conclusions

The findings of this study highlight the complexity of how and when different types of social support are exchanged on the QuitNow community forum. These findings provide directions for how social support can be more strategically employed and leveraged in these online contexts to support smoking cessation. Both community forum end-users and service providers would benefit from understanding the nuanced support needs of those trying to quit smoking.

## Appendix

Multimedia Appendix 1 Social support framework.
