# Supplementary material for: Assessment of Social Support and Quitting Smoking in an Online Community Forum: Study Involving Content Analysis
Source: JMIR Form Res. 2022 Jan 13;6(1):e34429. doi: 10.2196/34429 (PMC8796047; doi:10.2196/34429)
Supplement: Multimedia Appendix 1 [file formative_v6i1e34429_app1.docx]

**Multimedia Appendix 1: Social support framework**

| Code | Subcode | Description |
| --- | --- | --- |
| Emotional | *Confidentiality* | Promises to keep the recipient's problem in confidence. |
|  | *Encouragement* | Provides the recipient with hope and confidence. |
|  | *Listening* | Attentive comments as the recipient speaks. |
|  | *Physical affection* | Offers physical contact, including hugs, kisses, handholding, shoulder patting. |
|  | *Prayer* | Prays with the recipient. |
|  | *Relationship* | Stresses the importance of closeness and love in relationship with the recipient. |
|  | *Sympathy* | Expresses sorrow or regret for the recipient's situation and distress. |
|  | *Understanding/ empathy* | Expresses understanding of the situation or discloses a personal situation that communicates understanding. |
| Esteem | *Compliment* | Says positive things about the recipient or emphasizes the recipient's abilities. |
|  | *Relief of guilt* | Tries to alleviate the recipient's feelings of guilt about the situation. |
|  | *Validation* | Expresses agreement with the recipient's perspective on the situation. |
| Informational | *Referral* | Refers the recipient to some other source of help. |
|  | *Situation appraisal* | Reassesses or redefines the situation. |
|  | *Suggestion/advice* | Offers ideas and suggests actions. |
|  | *Teaching* | Provides detailed information, facts, or news about the situation or about skills needed to deal with the situation. |
| Network | *Access* | Offers to provide recipient with access to new companions. |
|  | *Companionship* | Reminds the person of availability of companions, of others who are similar in interests or experience. |
|  | *Presence* | Offers to spend time with the person, to be there. |
| Tangible | *Active participation* | Offers to join the recipient in action that reduces the stress. |
|  | *Direct task* | Offers to perform a task directly related to the stress. |
|  | *Indirect task* | Offers to take over one or more of the recipient's responsibilities while the recipient is under stress. |
|  | *Loan* | Offers to lend the recipient something (including money). |
|  | *Willingness* | Expresses willingness to help. |
| Other | *Appreciation* | Thanking posters for something. |
|  | *Happy holidays* | Wishing happy holidays to users. |
|  | *Welcoming* | Welcoming a poster to the forum. |
